# Neuropsychiatric manifestations inaugurating Biermer’s disease

**DOI:** 10.1192/j.eurpsy.2024.1294

**Published:** 2024-08-27

**Authors:** S. Bader, N. Msadek, S. Ellini, R. Damak, M. Cheour

**Affiliations:** Ibn El Omran psychiatry department, Razi Hospital, Mannouba, Tunisia

## Abstract

**Introduction:**

Vitamin B12 deficiency gives rise to a wide spectrum of hematological, gastrointestinal, psychiatric, and neurological disorders. Notable among the neuropsychiatric symptoms are mood disturbances, cognitive decline, and psychotic manifestations.

**Objectives:**

We present a case of a woman with neuropsychiatric symptoms linked to vitamin B12 deficiency to highlight certain organic aetiologies with psychiatric symptoms in the foreground.

**Methods:**

We discussed through a clinical case and a literature review, the relationship between neuropsychiatric symptoms and vitamine B12 deficiency in the context of biermer’s disease.

**Results:**

We presented a patient aged 51-years-old without neurological or psychiatric history, she was hospitalised in a psychiatry department for behavioral disturbances, hetero-aggression, and incoherent speech. The psychiatric examination revealed distant contact, inappropriate affects, disorganized speech with persecutory delusions, memory problems, and poor insight. Neurological et physical examinations were normal, and cerebral magnetic resonance imaging (MRI) showed no abnormalities. First, haloperidol 25mg was prescribed, however, there was only partial improvement. Complete blood counts revealed macrocytic anemia (Hemoglobin: 8 g/dL, mean corpuscular volume: 106 fL). Her serum B12 assay was 48.19 pmol/L.Given these results we proceed to a Fundic biopsy, performed by fibroscopy, that revealed fundic atrophy and intestinal metaplasia compatible with Biermer’s disease. Vitamin B12 replacement therapy began with hydroxocobalamin at 1000 μg/day intramuscularly for 15 days, followed by 1000 μg every 15 days for one month. Subsequently, there was a remarkable improvement in psychotic symptoms and cognitive function. Follow-up assessments demonstrated a return to baseline functioning.

**Conclusions:**

This case, coupled with prior studies, emphasizes the importance of considering vitamin B12 deficiency in the differential diagnosis of neuropsychiatric symptoms. Therefore, prompt diagnosis and treatment of vitamin B12 deficiency are imperative in preventing potential irreversible neurological damage.

**Disclosure of Interest:**

None Declared

